# Mobility Analysis of AmpuTees (MAAT I): Quality of life and satisfaction are strongly related to mobility for patients with a lower limb prosthesis

**DOI:** 10.1177/0309364617736089

**Published:** 2017-10-08

**Authors:** Shane R Wurdeman, Phillip M Stevens, James H Campbell

**Affiliations:** 1 Department of Clinical and Scientific Affairs, Hanger Clinic, Austin, TX, USA; 2 School of Allied Health Sciences, Baylor College of Medicine, Houston, TX, USA; 3 School of Medicine, University of Utah, Salt Lake City, UT, USA

**Keywords:** MAAT, amputation, mobility, quality of life, prosthetics

## Abstract

**Background:**

While rehabilitation professionals are historically trained to place emphasis on the restoration of mobility following lower limb amputation, changes in healthcare dynamics are placing an increased emphasis on the limb loss patient’s quality of life and general satisfaction. Thus, the relationship between these constructs and mobility in the patient with lower limb loss warrants further investigation.

**Objectives:**

To determine the relationship between mobility of the patient with lower limb loss and both (1) general satisfaction and (2) quality of life.

**Study design:**

Retrospective chart analysis.

**Methods:**

A retrospective chart review of the Prosthetic Limb Users Survey of Mobility and the Prosthesis Evaluation Questionnaire—Well-Being subsection. Pearson correlations were used to test relationships.

**Results:**

Data from 509 patients with a lower limb prosthesis were included. Mobility was found to be positively correlated with quality of life (*r* = 0.511, *p *< 0.001, 95% confidence interval (0.443, 0.569)) and general satisfaction (*r* = 0.475, *p* < 0.001, 95% confidence interval (0.403, 0.542)), as well as their arithmetic mean (i.e. Prosthesis Evaluation Questionnaire—Well-Being) (*r* = 0.533, *p *< 0.001, 95% confidence interval (0.466, 0.592)).

**Conclusion:**

This study provides evidence of a strong positive correlation between mobility and both quality of life and general satisfaction. Thus, in the holistic care of a patient with lower limb loss, maximizing mobility would correlate with greater quality of life and general satisfaction.

**Clinical relevance:**

There is growing emphasis on the quality of life and general satisfaction experienced by patients undergoing prosthetic rehabilitation. The results of this study underscore the importance of providing prosthetic rehabilitation that maximizes the patient’s mobility, noting that these individuals also report greater quality of life and general satisfaction.

## Background

Lower limb amputation is considered a major health event that can negatively impact a person’s functional mobility.^1,2^ Restoring functional mobility following lower limb amputation should be considered a primary goal of the rehabilitation process.^3,4^

In addition to the restoration of functional mobility, a second, more general rehabilitation goal is optimizing the person’s quality of life and satisfaction. Prosthetists, physical therapists, and physiatrists are trained with a mindset of providing a patient with a tool (i.e. the prosthesis) and training the patient to use that tool in order to improve mobility. This process is undertaken in the belief that it positively impacts a patient’s life, with expectations of improved quality of life and satisfaction. The objectives of enhanced mobility, quality of life and satisfaction appear to be closely related, yet their relationships either have not been clearly reported or have been confined to limited population sizes where such relationships were not the primary objective of the studies.

Suckow et al.^5^ investigated the impact of mobility for individuals with lower limb amputation on the construct of quality of life. This was done through a series of focus group interview sessions with 26 individuals of varying amputation etiologies, levels of amputation, and age. Among the participants, 65% felt mobility, or the lack thereof, had an impact on their quality of life. In another study, Norvell et al.^6^ reported a significant association between mobility and satisfaction with life in a group of 75 individuals with lower limb loss. In one of the larger studies, Pell et al.^7^ investigated 149 individuals with a major lower limb amputation assessing various aspects of quality of life using the Nottingham Health Profile questionnaire.^8^ Following stepwise logistic regression, mobility was found to be the only component that differed significantly between individuals with limb loss and non-amputee controls. In a recent systematic review, Davie-Smith et al.^9^ looked at various factors impacting quality of life for individuals with lower limb amputation due to peripheral arterial disease. Notably, the ability to walk successfully with a prosthesis was reported to have the greatest positive impact on quality of life. This led the authors to conclude the ability to walk with a prosthesis is of primary importance toward improving quality of life for this patient population.

This study is the first within a series of mobility analyses of amputees (MAAT) utilizing retrospective chart review of outcomes data being collected for patients with lower limb prostheses. The purpose of this study was to further establish the relationship between mobility and both quality of life and satisfaction for patients with lower limb amputation by examining a large group of diverse patients in a retrospective chart review. Based on the limited studies available,^5,7,9^ it was hypothesized that mobility would be positively correlated with quality of life. Additionally, based on the findings of Norvell et al.,^6^ it was further hypothesized that mobility would be strongly correlated with a patient’s general satisfaction with their situation.

## Methods

### Study design

In the first of the MAAT initiative, we performed a multi-site, retrospective review of outcomes data collected within a large, multi-site prosthetics provider. A convenience sample of the most recent 550 patients were extracted for analysis. The target goal was 500 patients with an expectation of 10% of data dropped due to incomplete information or not meeting inclusion criteria. For patients with multiple outcome data sets on file, only the most recent data were considered to eliminate patient duplication. Patients with incomplete outcome data were excluded. This retrospective database review was approved by Western Investigational Review Board (Protocol #20170059).

### Participants

Individuals with unilateral and bilateral lower limb amputation were included. Individuals were required to be the following: (1) age 18 or older, (2) present with amputation level/s of ankle disarticulation, transtibial, knee disarticulation, transfemoral, or hip disarticulation/hemipelvectomy, (3) currently using a prosthesis, and (4) should have the ability to read, write, and understand English or Spanish. There were no restrictions with regard to prosthetic device or Medicare Functional Classification Level (MFCL). MFCL is a United States–based classification system whereby all lower limb prosthesis users are categorized into four distinct classifications based on current and potential function. These classifications provide broad guidance for payment for services for prostheses by Medicare and are also utilized by most major third-party payers.^3^

### Procedure

Patients were asked to complete a self-report survey outcomes packet as part of their routine prosthetic care. Administration of the outcomes packet occurred at various points in a given subject’s prosthetic rehabilitation, including during a follow-up appointment with a legacy prosthesis, at the patient’s initial evaluation appointment for a replacement prosthesis or during the transition of a major prosthetic component such as the socket, foot, or knee. The outcomes packet includes the 12-item Prosthetic Limb Users Survey of Mobility (PLUS-M)^3,10,11^ 1 and the well-being subsection of the Prosthesis Evaluation Questionnaire (Prosthesis Evaluation Questionnaire—Well-Being (PEQ-WB)).^12–15^ The 12-item PLUS-M is a patient-reported outcome measure that asks individuals to rate the level of difficulty they experience across 12 different mobility tasks. Patients provide responses to the varying tasks reflecting five levels of ease: (1) Unable to do, (2) With much difficulty, (3) With some difficulty, (4) With a little difficulty, and (5) Without any difficulty. Each response is graded with its associated score (1–5). The summed score of all responses are then cross-referenced to a standardized *t*-score.^16^ This conversion facilitates comparison to a reference population as a *t*-score has a mean of 50 and a standard deviation of 10 points. For PLUS-M surveys that were missing a response, the appropriate scoring procedure was used as outlined by the instrument authors.^16^ Evidence of validity and reliability have been established for use of the PLUS-M in assessing self-rated mobility in patients with lower limb amputation.^3,16^

The PEQ-WB was originally published as a subsection of the larger Prosthesis Evaluation Questionnaire (PEQ).^12^ The complete PEQ is long and the extensive time required for administration and scoring make it prohibitive in the clinical setting.^12^ The PEQ-WB subsection, however, comprises only two questions that ask the patient to reflect on satisfaction and quality of life over the past 4 weeks. Specifically, the patient is asked to rate how satisfied they have been with how things have worked out since their amputation (i.e. general satisfaction) and to rate their quality of life. Notably, although the label “well-being” was attached to this subsection when first published, the current definition of “well-being” from organizations such as Health People 2020 places “well-being” as a sub-domain under quality of life.^17^ Furthermore, satisfaction is a type of positive emotion a patient holds about their life that contributes to well-being among other emotions. As a result, this study does not attempt to investigate the construct of well-being, but rather, the issues of quality of life and satisfaction. However, as it is the PEQ-WB composite score (comprising the mean of the scores for quality of life and satisfaction) that has been found to have evidence of validity and reliability, this score was also correlated with mobility.

Initially, the PEQ and PEQ-WB were published as continuous visual analog scales. However, for ease of administration and subsequent scoring, various subsections of the PEQ have been administered in the format of 5- and 10-point ordinal scales.^18^ As a standard of practice in participating clinics in this retrospective analysis, the PEQ-WB was administered in the format of a 10-point ordinal scale to allow for increased resolution over a 5-point ordinal scale. Notably, this concession to increase the ease of administration may have decreased resolution compared to a visual analog scale. Evidence of validity has been established for measuring both quality of life and satisfaction with the PEQ-WB in patients with lower limb amputation,^19^ and the PEQ is considered among the most commonly utilized survey instruments in prosthetics research.

There are many outcome measures available to measure quality of life and general satisfaction, and many of these are more detailed and informative than the PEQ-WB questions. However, these instruments require increased time to administer and score compared to the PEQ-WB. As these questionnaires were implemented as part of standard of delivery of care, there was great emphasis placed on clinical feasibility with priority given to brevity when selecting these outcomes instruments. Specifically, it is felt that as an additional task for clinicians in their busy work routine, any substantial time commitment would decrease the administration rates of the outcome measure.

### Analysis

The relationships between PLUS-M *t*-scores, satisfaction, quality of life, and PEQ-WB scores were investigated using separate Pearson product moment correlations. For all correlation analyses, the 95th percentile confidence interval was determined through the implementation of a bootstrapping procedure with 1000 iterations. Correlation coefficient effect sizes were classified according to Cohen’s recommendations.^20,21^ All statistical analyses were done in SPSS v20.

## Results

A convenience sample of the most recent 550 patients from participating clinics with completed outcome data sets was extracted. The patient demographic data were then checked at time of evaluation to confirm inclusion criteria. This resulted in dropping nine patients which were under age 18. For each ordinal score of the PEQ-WB, PLUS-M mobility *t*-scores were tabulated, and any data points outside the 95% tolerance interval based on the ordinal score’s mean were noted as outliers, resulting in an additional dropping of 32 data points. This resulted in 509 patients for which correlations were analyzed (Table 1).

**Table 1.** Study cohort characteristics.

Total patients included	509
Male	363
Female	146
Age (years)	
Mean	56.4
SD	14.6
Range	17–95
Height (cm)	
Mean	175.0
SD	14.1
Range	88.9–203.2
Mass (kg)	
Mean	91.9
SD	24.2
Range	42.2–199.6
Etiology (# of patients)	
Vascular disease/diabetes	202
Injury/trauma	163
Infection (without diabetes)	48
Cancer/tumor	23
Congenital/birth	22
Other	30
Unknown	21
Amputation level (# of patients)	
BK/Symes	350
AK/KD	108
PFA	1
Bilateral	53
AK-BK	8
AK-AK	4
BK-PFA	4
AK-PFA	1

SD: standard deviation; BK: below knee; AK: above knee; KD: knee disarticulation; PFA: partial foot amputation.

**Figure 1.**
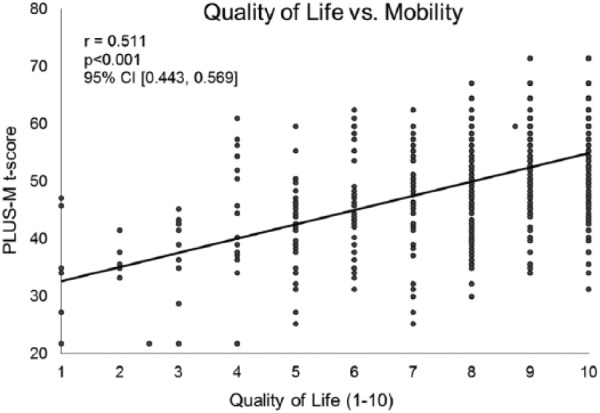
For patients with a lower limb prosthesis there is a strong and significant, positive relationship between quality of life and mobility. Quality of life was measured through the component question of the Prosthesis Evaluation Questionnaire—Well-Being subsection (PEQ-WB). Patient mobility was assessed via the Prosthetic Limb Users Survey of Mobility (PLUS-M). The positive relationship would indicate the individuals that are more mobile enjoy a higher quality of life (*n* = 509).

**Figure 2.**
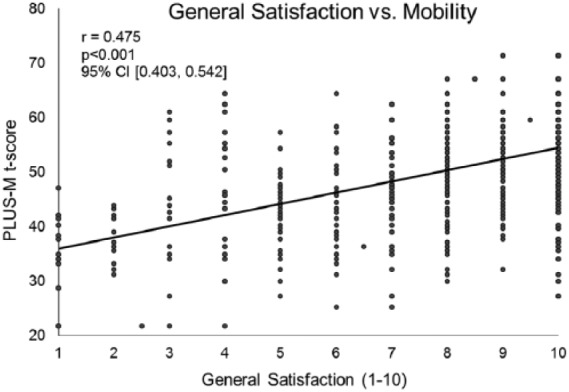
For patients with a lower limb prosthesis, there is a significant, positive relationship between their general satisfaction and mobility. These findings indicate the individuals that are more mobile generally report greater satisfaction with regard to how things have worked out since their amputation. Satisfaction was measured through the component question of the Prosthesis Evaluation Questionnaire—Well-Being subsection (PEQ-WB). Patient mobility was assessed via the Prosthetic Limb Users Survey of Mobility (PLUS-M) (*n* = 509).

**Figure 3.**
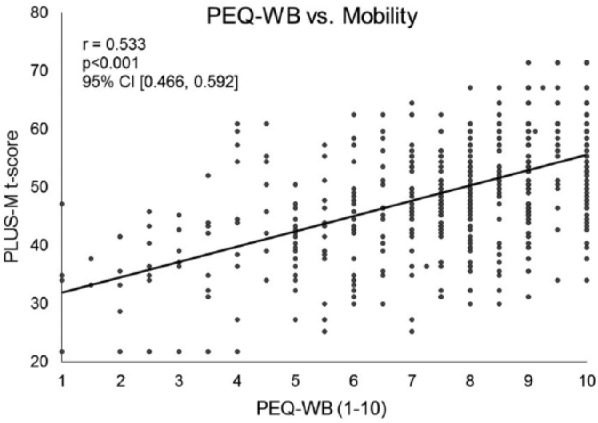
For patients with a lower limb prosthesis, there is a strong, significant relationship between the Prosthesis Evaluation Questionnaire—Well-Being subsection (PEQ-WB) and mobility. The positive relationship indicates patients reporting greater mobility tend to report higher quality of life and satisfaction. The PEQ-WB comprises the arithmetic mean of individual questions of quality of life and satisfaction and has been previously validated to be administered separate from the entire PEQ. Mobility was measured through the Prosthetic Limb Users Survey of Mobility (*n* = 509).

Quality of life and satisfaction were examined separately. Quality of life was significantly and positively correlated with the patient’s PLUS-M *t*-score (*r* = 0.511, *p *< 0.001, 95% confidence interval (CI) (0.443, 0.569); Figure 1). This was noted to be a strong correlation by Cohen standards.^20,21^ Similarly, general satisfaction with how things have worked out with regard to the patient’s amputation over the past 4 weeks was also significantly and positively correlated with the individual’s PLUS-M *t*-score (*r* = 0.475, *p* < 0.001, 95% CI (0.403, 0.542); Figure 2). This is referenced as a moderate correlation by Cohen standards.^20,21^ The score for the PEQ-WB was significantly and positively correlated with the patient’s PLUS-M *t*-score (*r* = 0.533, *p *< 0.001, 95% CI (0.466, 0.592); Figure 3). This is noted to be a strong relationship by Cohen standards.^20,21^

## Discussion

The objective of this study was to determine the relationship between self-reported mobility, and both quality of life and satisfaction for patients with a lower limb amputation. The results of this study confirm the initial hypothesis that there is a positive relationship between both of these constructs and mobility.

The emphasis of prosthetic rehabilitation has historically centered on mobility. By contrast, quality of life and satisfaction have been less recognized. Encouragingly, changes in healthcare policies appear to be placing an increased emphasis on patient’s quality of life and satisfaction, as evidenced by the launch of the Patient Centered Outcomes Research Institute by the enactment of the United States Affordable Care Act of 2010^22^ and the funding of the National Institutes of Health Patient-Reported Outcomes Measurement Information System (PROMIS).^23^ Both of these actions have increased the focus on measuring patient-reported outcomes including satisfaction and quality of life.

The close relationship between these factors and mobility should be fully appreciated in patients with a lower limb amputation. In this study, the significant relationships between mobility and both quality of life and satisfaction highlight the importance of maximizing mobility in patients with lower limb loss, both for the associated immediate functional benefits as well as its influence on the generalized domains of both quality of life and satisfaction in this population.

Quality of life and general satisfaction are multi-dimensional with individuals valuing these various dimensions at different levels. Mobility appears to explain a high percentage of the variability associated with quality of life, general satisfaction and their arithmetic mean, the PEQ-WB subsection score, with observed coefficients of determinations of 26.1%, 22.6%, and 28.4%, respectively.

During post-amputation rehabilitation, it is the role of the treating clinical team to identify those modifiable factors that may improve the patient’s quality of life and satisfaction. The maximization of mobility appears to be a significant consideration in this responsibility. Studies such as this one can support the relationships between key outcome metrics and those considerations identified as primary goals in rehabilitation. Future work is needed to expand this analysis to investigate other potential factors influencing quality of life and satisfaction for the patient with lower limb amputation.

### Study limitations

A strength of this study was its large study population. Studies in the domain of prosthetic rehabilitation rarely have sample sizes greater than 100 and are typically less than 40. The large sample size in this study (*n* = 509) reinforces that the findings represent the entire population of lower limb prosthesis users, reduce impact of individual variance, and increase statistical power to find significant results above and beyond individual variance or residual error. However, there are limitations. Specifically, as a retrospective analysis of outcomes data collected at multiple clinic sites at varying regions across the country, our results may overlook geographic or cultural variations related to mobility and quality of life that exist in local regions. Furthermore, having multiple clinicians involved in data collection introduces the chance for error. To minimize this limitation, clinicians were trained via face-to-face training sessions as part of the outcomes collection procedure. Additionally, our sample only included one individual with only a partial foot amputation, potentially limiting generalizability to these individuals. Finally, there are alternate ways of measuring quality of life and general satisfaction that may be more detailed and informative than the PEQ-WB questions. However, these alternate questionnaires are also more time-consuming and may have undermined clinician participation.

## Conclusion

Functional mobility is compromised in individuals dealing with lower limb loss. While prosthetic rehabilitation has traditionally placed large emphasis on improving and maximizing mobility, more recently rehabilitation has started to focus on both the quality of life and general satisfaction of the affected individuals.^22,23^ This study provides evidence that mobility is positively related to both considerations. Thus, in the holistic care of a patient with lower limb loss, maximizing mobility should be considered a primary goal.
